# Macrophage-derived HIV-1 carries bioactive TGF-beta

**DOI:** 10.1038/s41598-019-55615-8

**Published:** 2019-12-13

**Authors:** Anush Arakelyan, Jennifer D. Petersen, Jana Blazkova, Leonid Margolis

**Affiliations:** 1 Section on Intercellular Interactions, Eunice Kennedy-Shriver National Institute of Child Health and Human Development, National Institutes of Health, Bethesda, MD USA; 2 Section on Integrative Biophysics, Division of Basic and Translational Biophysics, Eunice-Kennedy National Institute of Child Health and Human Development, National Institutes of Health, Bethesda, MD USA; 3 Laboratory of Immunoregulation, National Institute of Allergy and Infectious Diseases, National Institutes of Health, Bethesda, MD USA

**Keywords:** Pathogens, HIV infections

## Abstract

Infected T cells and macrophages are the main producers of HIV-1 in infected individuals. Upon release from infected cells, HIV-1 incorporates various cellular membrane proteins, some of which are specific for these cells. However, the functions of cell-encoded proteins in virions remain largely unknown. We performed flow virometry to identify, in plasma of HIV-infected individuals, macrophage- and T-cell-derived HIV-1 virions, using cell-specific markers CD36 and CD27, respectively. Using four different methods, we demonstrated that CD36 on virions binds the immunosuppressive cytokine transforming growth factor beta (TGF-β) through a ligand, thrombospondin one (TSP-1). Flow virometry of individual virions showed that TGF-β was present on CD36+ virions (average, 28.2% ± 6.6% (*n* = 3)) but not on CD27+ virions (average, 1% ± 0.1% (*n* = 3)). TGF-β molecules present on captured CD36+ virions were biologically active, as evaluated with a reporter cell line. Delivery of TGF-β on HIV virions to HIV target cells may affect them, playing a significant role in viral pathogenesis.

## Introduction

HIV-1 virions released by productively infected cells, predominantly T cells and macrophages, contain not only virus-encoded proteins but also some cellular proteins^1–4^. The functions of the majority of cell-encoded molecules incorporated into virions, unlike those of virus-encoded molecules, are mostly not known.

Here, we investigated a function of macrophage-specific molecule CD36, which is incorporated into macrophage-derived HIV-1 virions. This protein distinguishes macrophage-derived virions from T-cell-derived virions, which incorporate CD27 into their membrane^3,4^.

CD36 belongs to the class B scavenger receptor family of plasma membrane proteins. In cells, it binds various ligands, in particular thrombospondin one (TSP-1)^5–7^, a major activator of anti-inflammatory cytokine transforming growth factor beta (TGF-β)^8,9^. TGF-β is released by cells in inactive form (L-TGF-β) bound to a latency-associated peptide (LAP), and its activation is mediated by binding to TSP-1^10,11^.

In contrast with the role of CD36 in cells, its function in HIV-1 virions has not been identified. Here, using single-virion analysis (“flow virometry”^12^), we found that CD36^+^ virions isolated from plasma of HIV-infected individuals are associated with TSP-1 and carry biologically active TGF-β.

## Results

**Figure 1:**
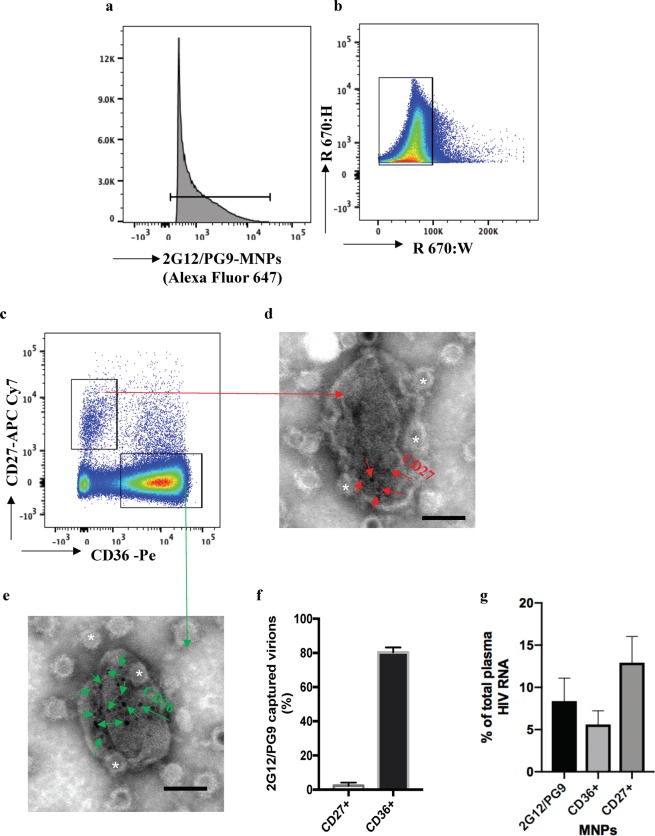
CD36 and CD27 on HIV-1 virions in blood plasma of infected individuals. (**a**) Gating strategy: threshold trigger setting on fluorescence height of MNP label Zenon Alexa 647. (**b**) Single gate defined by fluorescence height and fluorescence width, which excludes aggregates^12^. (**c**) Flow virometry of 2G12/PG9-captured HIV-1. Populations of CD27^+^ and CD36^+^ virions are presented. (**d**,**e**) CD27^+^ and CD36^+^ on HIV-1 virions captured with 2G12/PG9-MNPs (white asterisks). CD27 (red arrows) (**d**) and CD36 (green arrows) (**e**) are revealed by 6-nm colloidal gold particles. (**f**) Fractions of CD36^+^ and CD27^+^ among 2G12/PG9-captured virions as evaluated with flow virometry. Presented are means ± s.e.m. (*n* = 3). (**g**) HIV-1 RNA in fractions of virions captured with 2G12/PG9-MNPs, anti-CD36-MNPs, and anti-CD27-MNPs among total blood plasma virions. Presented are means ± s.e.m. (*n* = 3).

We applied flow virometry^12,13^ to analyze cellular proteins on HIV-1 in blood plasma of infected individuals. We coupled 15-nm iron oxide magnetic nanoparticles (MNPs) with two anti-Env antibodies: 2G12, which recognizes high-mannose clusters on gp120 and binds to monomers, dimers, and trimers of gp120, and PG9, which recognizes mostly trimeric forms of gp120^14,15^. To visualize MNP–antibody complexes, we stained them with fluorescent goat anti-human Fab fragments (Fig. 1a,b). The labeled anti-HIV MNPs were incubated with HIV+ blood plasma. Virions captured by these MNPs were further stained with antibodies against CD27 and CD36. These stained MNP-captured virions were retained on magnetic columns, eluted from the column, and analyzed with a flow cytometer (Fig. 1a–c).

To evaluate the amounts of these virions, i.e., those with Env recognized by PG9/2G12 in the total pool of plasma HIV, we quantified them by measuring viral RNA using the Cobas Ampliprep/Cobas Taqman HIV-1 Test. We found that virions carrying Env with a conformation recognized by 2G12/PG9 constituted 8.3% ± 2.7% (*n* = 3) of total viral RNA in plasma (Fig. 1g).

We further analyzed virions captured with PG9/2G12-MNPs for the presence of CD27 and CD36 using fluorescent antibodies against these cellular molecules. Flow analysis revealed that among these captured virions on average 2.3% ± 1.8% (*n* = 3), were positive for CD27 and all of them were negative for CD36, whereas 80.2% ± 3% (*n* = 3) were positive for CD36 and negative for CD27 (Fig. 1f). We confirmed the presence of CD27 and CD36 on captured virions using negative stain immunogold electron microscopy (Fig. 1d,e). To visualize these cellular molecules, we used 6-nm colloidal gold-conjugated goat anti-mouse secondary antibodies. On average, on captured virions there were 6.6 ± 1.2 (*n* = 24) gold particles revealing CD27 (Fig. 1d), and 22.2 ± 2.7 (*n* = 25) particles revealing CD36, bar: 50 nm (Fig. 1e).

To evaluate the sizes of CD36^+^ and CD27^+^ viral fractions in the *total* pool of plasma HIV rather than in the viral subset recognized by PG9/2G12 antibodies, we captured virions with MNPs coupled to anti-CD36 or anti-CD27 antibodies. We again quantitated the amounts of viruses in each of these fractions using the Cobas Ampliprep/Cobas Taqman HIV-1 Test. CD36^+^ virions, i.e. captured with anti-CD36 MNPs constituted 5.6% ± 1.6% (*n* = 3), while CD27^+^ virions, i.e. captured with anti-CD27 MNPs constituted 9.9% ± 4.6% of total plasma virus (Fig. 1g).

**Figure 2:**
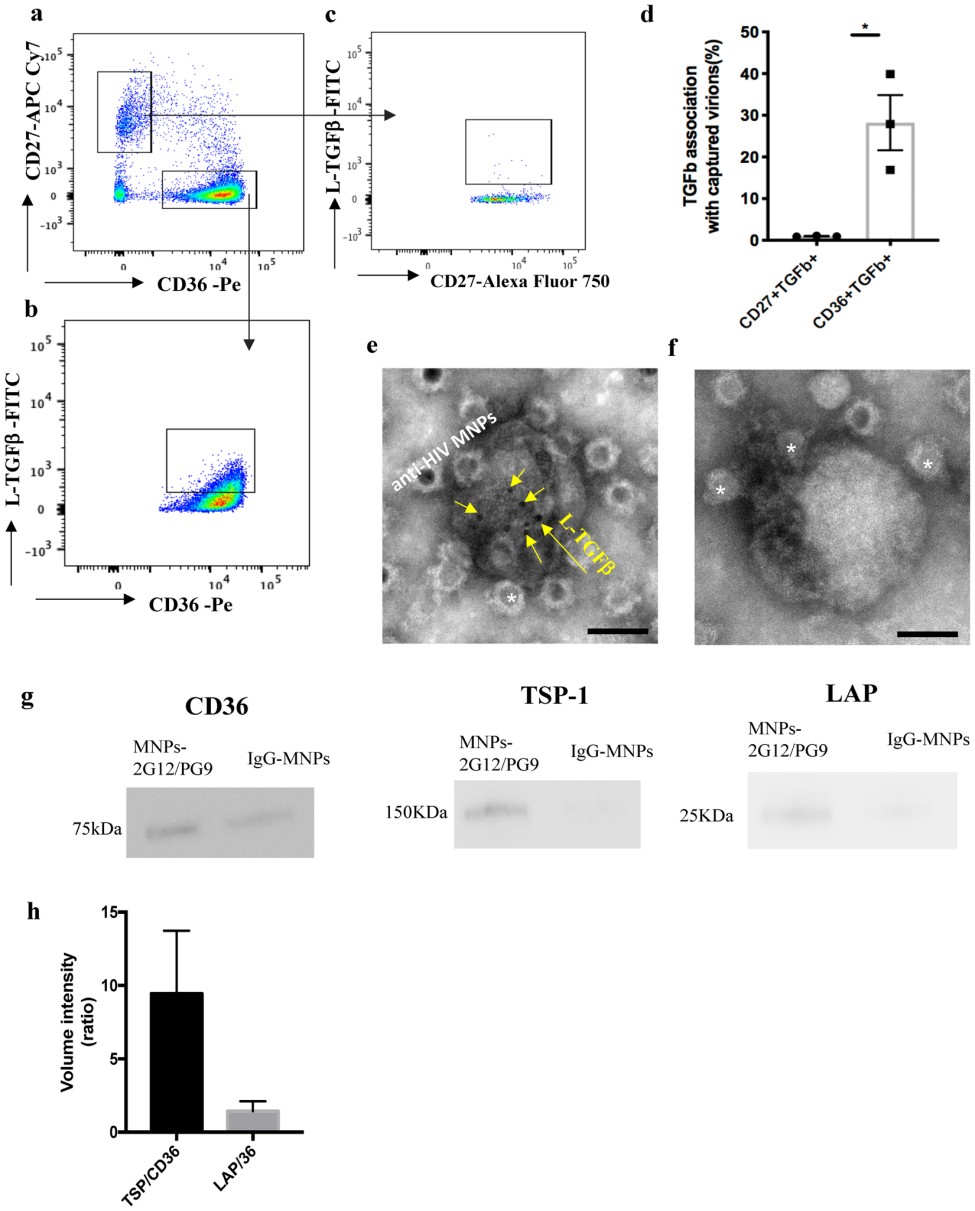
Visualization of TGF-β on HIV-1 virions. (**a**) Flow virometry of virions from blood plasma of infected individual gated on CD36 and CD27. (**b**) TGF-β^+^ virions in the population of CD36^+^ virions. (**c**) The lack of TGF-β^+^ virions in the population of CD27^+^ virions. A representative experiment out of three is presented. **(d**) Fractions of TGF-β^+^ on CD36^+^ and CD27^+^ virions captured with 2G12/PG9–MNPs as evaluated with flow virometry. Presented are means ± s.e.m. (*n* = 3). (**e**) L-TGF-β on virions captured with 2G12/PG9-MNPs. Electron micrograph of a negatively stained preparation. MNPs are marked with white asterisks; L-TGF-β is revealed by 6-nm colloidal gold-labeled secondary antibodies (yellow arrows). Bar: 50 nm (**f**) Control for specificity of staining. Virions captured with 2G12/PG9-MNPs and incubated with 6-nm colloidal gold-labelled secondary antibodies without primary anti L-TGF-β antibodies. MNPs are marked with white asterisks. Bar: 50 nm (**g**) CD36, TSP, and LAP proteins present in 2G12/PG9-MNP captured virions. Western blot analysis. A representative experiment out of three is presented. (For full-length gels/blots, see Supplementary Fig. 1). (**h**) Relative presence of TSP and LAP on captured virions. Average values from three experiments are presented.

In further study, we investigated whether the CD36 molecules on HIV-1 are associated with TGF-β, as has been shown to be the case with this molecule expressed on macrophages. Towards this goal, we captured virions with 2G12/PG9-MNPs, stained them for CD27 and CD36, and evaluated them for the presence of TGF-β by staining virions with antibodies against L-TGF-β (Fig. 2a–c). L-TGF-β was revealed on CD36^+^ virions (average, 28.2% ± 6.6% (*n* = 3)), but not on CD27+ virions (average 1% ± 0.1% (*n* = 3)); *p* = 0.03 (Fig. 2d).

The presence of L-TGF-β on captured virions was further confirmed with immunoelectron microscopy. Again, we captured virions with 2G12/PG9-MNPs and stained captured virions with anti-L-TGF-β antibodies. Colloidal gold-conjugated secondary goat anti-mouse antibodies were used to reveal the sites of binding of anti-L-TGF-β antibodies. Figure 2e demonstrates that L-TGF-β was clustered on viral particles. To quantify the proportion of virions bearing immunogold label for L-TGF-β, five randomly selected grid squares of a 200 mesh EM grid were searched. This area contained 53 virions, 17 of which carried immunogold label for L-TGF-β. On average, there were 8 ± 1.9 (*n* = 17) gold particles per labeled virion. Control experiments demonstrated the specificity of L-TGF-β visualization on virions (Fig. 2f).

We confirmed the presence of LAP on captured virions using a Luminex platform and Western Blot. To detect LAP with Luminex, we developed an assay using specific capture and detection antibodies. With this assay we revealed LAP in 2G12/PG9-MNP captured fractions at a concentration of 802.44 ± 247.4 pg/mL (*n* = 3). In addition, on 2G12/PG9-MNP captured virions Western blot revealed the presence of CD36 and TSP-1 (Fig. 2g). The amounts of these proteins varied from one plasma sample to another. However, the ratios of TSP-1/CD36 and LAP/CD36 had relatively small variability: 9.4 ± 4.2 (*n* = 3) and 1.4 ± 0.6 (*n* = 3), respectively (Fig. 2h), suggesting that these molecules are linked (correlation coefficient *r* = 0.98).

**Figure 3:**
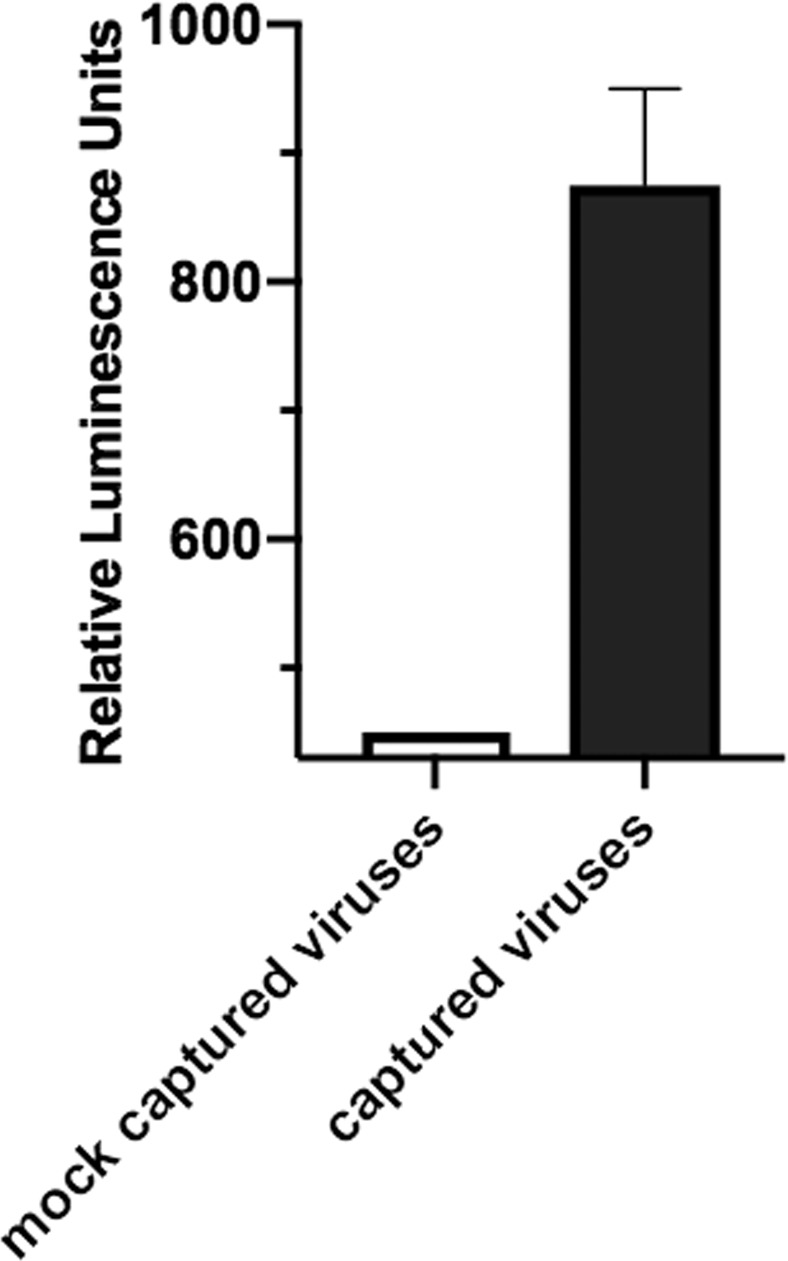
Bioactivity of HIV-associated TGF-β. Bioactivity was evaluated in TGF-β reporter NIH/3T3 cells treated with HIV-1 virions captured with 2G12/PG9-MNPs from blood plasma of infected individual or with non-specific IgG-MNPs incubated with blood plasma of infected individual. Presented is the luminescence of treated cells above the background fluorescence of control untreated cells (*n* = 2).

Finally, to investigate whether TGF-β molecules present on captured virions are biologically active, we incubated captured viruses with a reporter cell line that highly expresses TGF-β receptors. Binding of TGF-β activates transcription factors in these cells, as revealed by luciferase fluorescence^16^. Captured viruses from the two selected plasmas induced luciferase activity in the reporter cells, thus demonstrating that TGF-β molecules associated with these virions are biologically active. In control experiments where instead of 2G12/PG9-MNPs we used IgG-MNPs, no activation of transcription factors in the treated cells was observed (Fig. 3). Thus, in infected individuals, there are macrophage-derived HIV-1 that carry a bioactive TGF-β.

## Discussion

In this work, we focused on HIV-1 virions carrying macrophage-specific membrane molecule CD36. We found that these HIV virions may be of special importance, since some of them carry the immunosuppressive cytokine TGF-β. To identify such virions, we used flow virometry, a technology that allows us to characterize proteins on single viral particles by capturing them with 15-nm magnetic nanoparticles (MNPs) coupled to specific antibodies and staining these captured virions for the presence of other antigens^12^.

Here, we used two different strategies to capture virions: (i) with MNPs coupled to antibodies that recognize on virions cellular antigens, macrophage-specific CD36 or T-cell-specific CD27; (ii) with MNPs coupled to antibodies that recognize Env, 2G12/PG9. While with the first strategy we captured almost all virions^17^ that carry CD27 or CD36, with the second strategy we captured only those virions that carry Env in a particular conformation, since currently there is no antibody available that would recognize *all* conserved viral epitopes present on virions^18^. Specifically, PG9 binds to trimeric gp120, while 2G12 has a broader recognition ability and binds also to non-trimeric conformations of Env. Therefore, these two antibodies in combination capture virions carrying Env in various conformations which together constitute ~8% of the total viral population.

Capture of virions through CD27 or CD36 showed that more virions came from T cells. This result is in general agreement with the common notion that the main producers of HIV-1 are central memory T cells^19^. In contrast, among virions that carry Env in the conformation recognized by PG9/2G12 antibodies, the distribution of macrophage-derived and T-cell-derived virions was different. Among these PG9/2G12-captured virions, the majority (~80%) carry CD36. Thus, while the majority of virions in plasma come from CD4 T cells, rather than from macrophages, the population of virions with Env recognized by 2G12/PG9 antibodies is enriched with macrophage-derived CD36^+^ virions.

Importantly, the amounts of CD36^+^ virions as evaluated from two different capture strategies are in general agreement: 2G12/PG9-MNPs capture ~8% of total virions, of which 80% are CD36^+^; thus, according to these data these virions constitute approximately 6.4% (80% of 8%) of the total, while the CD36^+^ virions directly captured with anti-CD36 MNPs constitute 5.6% of the total.

We further focused on CD36^+^ virions, as we found that some of them are associated with TGF-β. TGF-β was found only on CD36^+^ virions but not on CD27^+^ virions isolated from blood plasma of infected individuals. We demonstrated this by means of four different methods: (i) flow virometry using fluorescent antibodies against CD36 and TGF-β demonstrated the presence of TGF-β on approximately a third of CD36^+^ virions; (ii) immunogold EM demonstrated the presence of TGF-β as well as CD36 on captured virions; (iii) Western blot assay revealed the presence of LAP; (iv) TGF-β was also revealed with a Luminex bead assay in the fraction of captured virions.

Does the association of TGF-β with CD36 through TSP-1 on HIV-1 virions mirror that on macrophages? In macrophages, it has been shown that CD36 on plasma membrane binds TSP-1, which interacts with the LAP of L-TGF-β, and that this interaction results in a conformational change that exposes the TGF-β receptor binding site on the L-TGF-β. This change transforms L-TGF-β to active TGF-β capable of binding to its receptor. In this model, LAP remained associated with the TGF-β after interacting with TSP-1^20^. Other mechanisms for L-TGF-β activation have been suggested^21^. Since this is the first report on the presence of TGF-β on virions, further studies should be performed to determine whether the other established mechanisms of activation on cells operate also for HIV-bound TGF-β. Here, by using Western blot, we found TSP-1, the main activator of TGF-β, in the fraction of captured virions carrying CD36. Binding of TGF-β to virions may occur in the course of virus biogenesis (e.g., in CD36-enriched virus-containing compartments^22,23^) or after virus is released from infected macrophages. Whichever is the exact mechanism of TGF-β binding to macrophage-derived HIV, on virions this cytokine remains bioactive, as shown by incubation of the captured virions with the reporter cells. TGF-β was shown in different biological systems to have strong immunomodulating effects^24,25^. It is conceivable that this bioactive cytokine carried on HIV-1 virions can change the immune status of the HIV targets. The exact effect of TGF-β carried on viral particles on the physiology of the HIV-target cells remains to be investigated.

Our study has several limitations: (i) MNPs coupled to 2G12 and PG9 antibodies capture a certain subset of virions^17^, while gp120 might exist in other conformations not recognized by these antibodies^15^; (ii) In our work we traced about 16% of total virions to their cells of origin as not all virions produced by T cells or macrophages carry respectively CD27 and CD36; (iii) Some of the plasma virions of interest might not have been captured, since their Env molecules could have been blocked by immunoglobulins generated *in vivo* during the course of infection; (iv) In our flow analysis, we might have underestimated the number of CD27^+^ virions, since electron microscopy showed that some of these virions carry only few CD27 molecules. Revealing CD27 on such virions might be below the limits of detection of flow virometry^17^.

In spite of these limitations, our work for the first time has demonstrated the existence of HIV-1 virions derived from macrophages that are associated with an important cytokine, TGF-β. This HIV-1-associated cytokine retains its biological activity and might play a role in viral pathogenesis affecting target cells.

## Materials and Methods

### Human subjects

Samples of blood plasma were obtained from three clade-C HIV-infected ART-naïve individuals in the framework of the HIV Pathogenesis Program of the Massachusetts General Hospital (MGH)/Howard Hughes Medical Institute, Boston and the University of KwaZulu-Natal, Durban, South Africa. Plasma collection was performed in accordance with relevant guidelines including patients informed consent and was approved by the MGH IRB (#2000P001150 & #2007P000473). Plasma samples were anonymized and the protocol was approved by the NIH Office of Human Subject Research (OHSRP #12395).

Viral loads as measured with the Taqman HIV-1 Test assay, Version 2.0 (Roche Diagnostics), were 174,328 C/mL, 274,395 C/mL, and 532,400 C/mL. The CD4^+^ T cell count was 433, 392 and 470 (average 431.7 ± 22.53).

### Flow virometry

We coupled 15-nm iron oxide nanoparticles (MNPs) (Ocean Nanotech) to 2G12 (Polymun Scientific) or PG9 (Polymun Scientific) according to the manufacturer’s protocol, resuspended them in 2 ml of wash/storage buffer, and stored the suspension at 4 °C until use. MNPs conjugated with antibodies were labeled with goat anti-human Fab fluorescent fragment (Zenon Invitrogen) for 30 minutes at room temperature. Labeled antibody-MNP complexes were washed on 100 K Nanosep (Pall) columns. Labeled antibody-MNP were incubated with 100 µL of blood plasma for 40 minutes at 37 °C; then the captured virions were stained with anti-CD27-Alexa Fluor 750 (clone: CLB-27/1; Invitrogen), anti-C36-Pe (5–271, Biolegend), and anti-L-TGF-β-FITC (clone: TW4-2F8; Biolegend) antibodies for an additional 20 min. After captured and stained virions were purified on magnetic columns attached to a magnet that generates a high magnetic field, the complexes were intensively washed with washing buffer (0.5% BSA and 2 mM EDTA in PBS), eluted from columns in 400 µL of PBS, and fixed in 2% PFA. We analyzed the complexes obtained on an LSR II (Becton Dickinson) flow cytometer equipped with 355-, 407-, 488-, 532-, and 638-nm laser lines by thresholding on fluorescence of MNP-antibody label. Compensation beads (BD) were used to perform compensation controls. We analyzed results using FlowJo software V.10.4.2 (Tri-Star). The efficiency of capture of specific virions with antibody-coupled MNPs was shown to be about 99%, and the sensitivity was sufficient to capture particular Env+ virions in plasma that contains 10^4^ RNA copies/mL^12,17^.

### Western blotting

We incubated 100 µL of blood plasma with a mix of 2G12/PG9-MNPs for 1 h at 37 °C. The mixture was washed on a magnetic column, eluted from the column, and resuspended in 100 µL of PBS. Captured virions were subjected to Western blot analysis. We extracted total proteins from captured fractions using RIPA Lysis and Extraction Buffer (Thermo Fisher Scientific, Waltham, MA). We loaded 10 µL of proteins on a 4%-20% precast polyacrylamide gel (Bio-Rad Laboratories, Hercules, CA), separated them with SDS-PAGE, and then transferred them to PVDF membranes and probed them with anti-CD36/SRB3 (1 μg/mL; R&D Systems), anti-TSP-1 (0.1 μg/mL; Millipore), and anti-LAP(TGF-β -1) (2 μg/mL; R&D Systems). Primary antibodies were revealed by anti-mouse IgG secondary antibodies (Bio-Rad Laboratories) or by anti-goat IgG secondary antibodies (R&D Systems) conjugated to horseradish (HRP) peroxidase. We detected peroxidase activity on digital images using V3 Western Workflow™ (Bio-Rad Laboratories, Hercules, CA). We analyzed the images using Image Lab 6.00.

### HIV RNA quantification

We quantified HIV virions from plasma- and MNP-captured fractions using the Cobas Ampliprep/Cobas Taqman HIV-1 Test, Version 2.0 (Roche Diagnostics). The percentages of 2G12/PG9-captured, CD36^+^, and CD27^+^ virions in plasma were calculated as a ratio of captured and total plasma virion-associated HIV RNA.

### Electron microscopy: negative stain of HIV with immunogold labeling

We inactivated MNP-purified HIV samples by fixing them with 2% paraformaldehyde (PFA) in PBS. For negative staining without immunogold labeling, freshly glow-discharged, formvar and carbon coated, 200 mesh copper grids (Electron Microscopy Sciences, EMS) were inverted on 5-µL drops of sample on Parafilm for 1 minute. Grids with adhered sample were transferred across two drops of syringe-filtered 2% aqueous uranyl acetate (UA) (EMS) for 1 minute, after which the grid was blotted with filter paper, allowing a thin layer of UA to dry on the grid.

Samples to be immunogold-labeled were adhered to freshly glow discharged, formvar and carbon coated, 200 mesh gold grids (EMS) and handled with non-magnetic forceps. After the samples were adhered, grids were passed through 20-µL drops of filtered PBS, and then incubated on drops of blocking solution containing 2% BSA (Sigma) in PBS for 30 minutes, to reduce non-specific antibody binding. Samples were covered during the incubation steps to prevent evaporation.

Primary antibody to CD36 (5–271; Biolegend), CD27 (clone: CLB-27/1; Invitrogen) or L-TGFβ-1 (clone: TW4-2F8; Biolegend) was diluted in filtered blocking solution to a working concentration of 20 µg/mL. After blocking, grids were blotted lightly with filter paper to remove excess solution before transfer to primary antibody droplets and then incubated for 60 minutes. Then, grids were transferred across two drops of blocking solution to rinse away unbound primary antibody and incubated for 20 minutes to block before incubation with secondary antibody. Secondary antibody (6-nm colloidal gold-conjugated Gt α Ms IgG; EMS) was diluted 1:25 in filtered blocking solution. Grids were lightly blotted before transfer to droplets of secondary antibody and incubated for 30 minutes and then rinsed in 3 drops of PBS. Prior to negative stain, grids were quickly transferred to a drop of distilled water to remove PBS before negative staining as described above. As a control for non-specific labeling with secondary antibody, primary antibody was omitted on control grids. in which case gold particles were only rarely observed.

We observed samples using a Technai T20 transmission electron microscope operated at 200 kV and collected images using an AMT NanoSprint1200 CMOS detector (Advanced Microscopy Techniques, Woburn MA).

### Multiplex-bead-array assay for L-TGF-β

Antibodies and cytokine standards were purchased as antibody pairs from R&D Systems (Minneapolis, MN). Individual Luminex magnetic bead sets (Luminex, Riverside, CA) were coupled to cytokine-specific capture antibodies anti- L-TGF-β (MAB246) according to the manufacturer’s recommendations. Conjugated beads were washed and kept at 4 °C until use. Biotinylated polyclonal antibodies were used at 100 ng/mL. All assay procedures were performed in assay buffer containing PBS supplemented with 1% normal mouse serum (GIBCO BRL), 1% normal goat serum (GIBCO BRL), and 20 mM Tris-HCl (pH 7.4). We ran the assays using 1,200 beads per well in a total volume of 50 µL. We added 50 µL in duplicates of 2G12/PG9–MNP-captured virions to the wells containing cytokine capture beads and incubated for 2 h on a shaker. Afterwards, the plate was washed on a magnetic washer twice with 200-µl assay buffer. The beads were then resuspended in 50 µL of assay buffer containing biotinylated polyclonal antibodies against L-TGF-β and incubated for 1 hour at room temperature. We washed plates again using a magnetic washer twice with 200 µL of assay buffer, and then added 50 µL of assay buffer containing a 1 µg/mL solution of streptavidin-PE (Molecular Probes, Eugene, OR) to each well. The plates were incubated for 45 minutes at room temperature, washed twice using a magnetic washer, and resuspended them in 100 µL of PBS. The plates were read on a Luminex-200 platform. For each bead, 100 beads per well were collected. The median fluorescence intensity of beads was recorded for each bead and was used for analysis with the Bio-Plex Manager software (version 4.0; Bio-Rad, Hercules, CA) using a 5 P regression algorithm. Values that were below the lower limit of detection (LLD) were reported as the midpoints between zero and the LLD (LLDs in pg/mL: L-TGF-β 4.7).

### TGF-β bioactivity assay

To test bioactivity of TGF**-**β in captured fraction stable SMAD/TGF-β reporter cell line (NIH/3T3#-SMAD) was used as a reporter system for monitoring the activation of SMAD triggered by TGF-β. 10^6^ cells/mL were washed with complete growth media, resuspended in 10 mL of complete growth media and used for bioactivity assay when reached 90% confluency.

Trypsinized cells were washed, seeded in 96 well white plate at 10^4^ cells in 100 µL per well in duplicates per condition and incubated overnight at 37 °C and 5% CO_2_. Next day, to remove not adhered cells media was aspirated from wells and virion captured fraction in 50 µl of media was added directly to the cells and incubated for 6 h/16 h.After removal of media cells were washed with PBS and incubated with 20 µL lysis buffer at room temperature for 15 min followed by addition of 100 µl of luciferase substrate and by reading the plate with Luminometer.

### Statistical analysis

We performed statistical analysis using the GraphPad Prism V7.0. Shapiro-Wilk normality test to test for normal distribution. The distribution was considered normal when *p* ≤ 0.05. *T-*tests were used to compare two groups at 95% confidence interval (CI). Differences were considered statistically significant when *p* ≤ 0.05.

## Supplementary information

Supplementary Figure 1
