# Supplementary material for: Macrophage-derived HIV-1 carries bioactive TGF-beta
Source: Sci Rep. 2019 Dec 13;9:19100. doi: 10.1038/s41598-019-55615-8 (PMC6911061; doi:10.1038/s41598-019-55615-8)
Supplement: Supplementary file 1 — Supplementary Figure 1 [file 41598_2019_55615_MOESM1_ESM.pdf]

Macrophage-derived HIV-1 carries bioactive TGF-beta

Short Title: TGF-b on HIV-1

Anush Arakelyan[1]*, Jennifer Petersen[2], Jana Blazkova[3], Leonid Margolis[1]*

[1]Section on Intercellular Interactions, and [2]Section on Integrative Biophysics
Eunice-Kennedy National Institute of Child Health and Human Development,
[3]Laboratory of Immunoregulation, National Institute of Allergy and Infectious Diseases
National Institutes of Health, Bethesda MD, USA

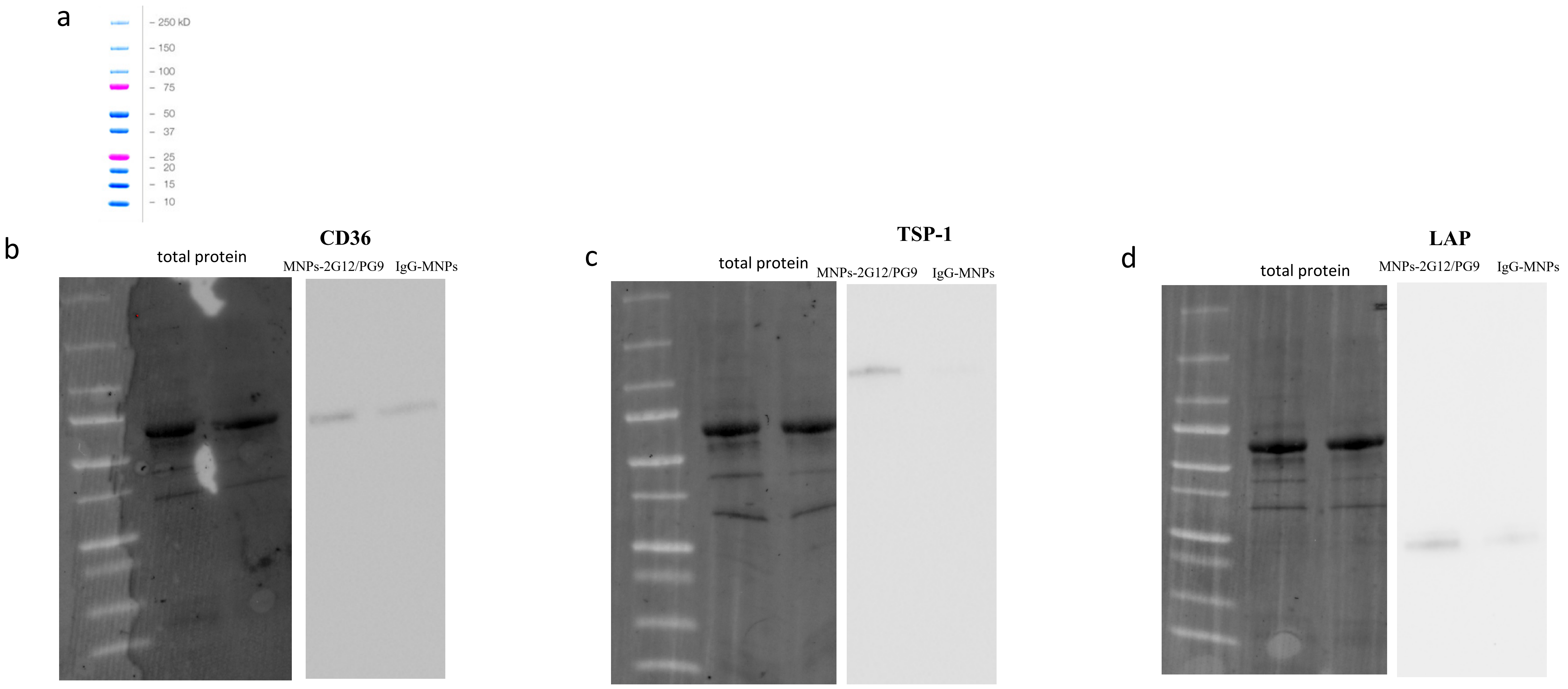

Supplementary Figure 1 (extended version of Fig. 2g)

a. Protein ladder used in Western blot experiments to estimate molecular size of the proteins.
b-d. Presented are full-length stain-free total protein gels (left panels) and blots (right panels) of CD36 **(b)**, TSP-1 **(c)**, and LAP **(d)** proteins present in 2G12/PG9-MNP -captured virions.
